# Evidence of Tau Hyperphosphorylation and Dystrophic Microglia in the Common Marmoset

**DOI:** 10.3389/fnagi.2016.00315

**Published:** 2016-12-22

**Authors:** Juan D. Rodriguez-Callejas, Eberhard Fuchs, Claudia Perez-Cruz

**Affiliations:** ^1^Laboratory of Neuroplasticity and Neurodegeneration, Department of Pharmacology, Center for Research and Advanced Studies (CINVESTAV)Mexico City, Mexico; ^2^Clinical Neurobiology Laboratory, German Primate Center – Leibniz Institute for Primate Research, GöttingenGermany

**Keywords:** aging, neurodegeneration, animal models, non-human primate

## Abstract

Common marmosets (*Callithrix jacchus*) have recently gained popularity in biomedical research as models of aging research. Basically, they confer advantages from other non-human primates due to their shorter lifespan with onset of appearance of aging at 8 years. Old marmosets present some markers linked to neurodegeneration in the brain such as amyloid beta (Aβ)_1-42_ and Aβ_1-40_. However, there are no studies exploring other cellular markers associated with neurodegenerative diseases in this non-human primate. Using immunohistochemistry, we analyzed brains of male adolescent, adult, old, and aged marmosets. We observed accumulation of Aβ_1-40_ and Aβ_1-42_ in the cortex of aged subjects. Tau hyperphosphorylation was already detected in the brain of adolescent animals and increased with aging in a more fibrillary form. Microglia activation was also observed in the aging process, while a dystrophic phenotype accumulates in aged subjects. Interestingly, dystrophic microglia contained hyperphosphorylated tau, but active microglia did not. These results support previous findings regarding microglia dysfunctionality in aging and neurodegenerative diseases as Alzheimer’s disease. Further studies should explore the functional consequences of these findings to position this non-human primate as animal model of aging and neurodegeneration.

## Introduction

Our world population is constantly increasing, and life expectancy in developing countries does as well (World Health Organization, 2015). Aging is characterized by an increasing morbidity and functional decline that eventually results in the death of an organism. Aging is the largest risk factor for several human diseases (López-Otín et al., 2013), and nowadays, cognitive decline has been observed to prevail in aged subjects (Hedden and Gabrieli, 2004). Currently, there are no treatments to cure or prevent cognitive decline due to aging or neurodegenerative diseases as Alzheimer’s disease (AD). Diverse animal models of aging have been developed to gain a better understanding of the biological causes of senescence, longevity, or disease (Woodruff-Pak, 2008; Yeoman et al., 2012). Animals have been mechanically drug-induced or genetically manipulated to model the aging process (Woodruff-Pak, 2008; Tomobe and Nomura, 2009; Bilkei-Gorzo, 2014; Mitchell et al., 2015). Likewise, invertebrates are also employed (Yeoman and Faragher, 2001; Woodruff-Pak, 2008; Chiu et al., 2011). These models have proven to be useful in the study of physiological processes related to aging and neurodegeneration. Nevertheless, non-human primates have gained popularity in aging research due to their ability to provide a better anatomical and pathophysiological representation of the human central nervous system (CNS; Scientific Committee on Health and Environmental Risks, 2009; Teo et al., 2012). Cognitive capabilities and memory task related to human conditions are more closely assessed in non-human primates than in other mammals (Huang et al., 2016). Moreover, the progression of human diseases is better reflected in non-human primates (Teo et al., 2012). Considerable research has focused on the Old World monkeys (Carlsson et al., 2004; Huang et al., 2016). However, for aging studies, smaller New World species, like the marmoset, are attractive candidates due to their shorter life span with onset of appearance of aging at 8 years (Abbott and Barnett, 2003) while a significant reduction in early adult mortality is seen in captivity where marmosets can live up to 16–18 years (Tardif et al., 2011). Common marmosets originated from the Amazon, and are characterized by a small body size (20–30 cm and 250–600 g), an accelerated life history (sexually mature at 1.5 years), and a capacity to produce high numbers of offspring (∼12 over 3 years from one female; Okano et al., 2012). In addition, there is a high genetic homology to humans (Ross et al., 2012). Therefore, marmosets are nowadays widely used for biomedical research (for review see t’Hart et al., 2012).

Aging in marmosets causes diverse alterations in the CNS (Tardif et al., 2011), such as decrease neurogenesis in the dentate gyrus of the hippocampal formation (Leuner et al., 2007), loss of calbindin positive cells in the basal forebrain (Wu et al., 2003), and cortical amyloid plaques (Geula et al., 2002). However, there is a lack of studies regarding the presence of neurodegenerative markers in this non-human primate.

In this study, we aimed to assess the presence of human neurodegenerative markers such as hyperphosphorylation of tau protein and activation of microglia cells. We have detected amyloid plaques in the cortex of aged subjects. An abnormally hyperphosphorylated tau protein was present already in adolescent marmosets, whereas those alterations were more pronounced with aging. Microglia phenotype differs across ages, as adult and old groups had abundant active microglia cells, while dystrophic cells increased significantly in aged animals compared to younger subjects. Importantly, tau hyperphosphorylation and aggregation were present only in dystrophic microglia cells. Thus, the presence of these neurodegenerative markers position marmosets as a potential model of neurodegeneration related to aging.

## Materials and Methods

### Subjects

Laboratory-bred common marmoset monkeys (*Callithrix jacchus*) were housed at the German Primate Center, Göttingen, Germany, under standard conditions complying with the European Union guidelines for the accommodation and care of animals used for experimental and other scientific purposes (2007/526/EC). All animal experiments were performed in accordance with the German Animal Welfare Act, which strictly adheres to the European Union guidelines (EU directive 2010/63/EU) on the use of non-human primates for biomedical research. Experienced veterinarians and caretakers constantly monitored the animals. The experiments were ethically approved by the Lower Saxony State Office for Consumer Protection and Food Safety (LAVES, Oldenburg, Germany). Animals did not present neurological disorders or other injuries that can cause trauma to the CNS.

### Tissue Preparation

Brains of male marmosets of different ages were used in the current study: two adolescent (A: mean age 1.6 years), two adults (Ad: mean age 5.5 years), five old (O: mean age 11 years), and two aged (Ag: mean age 18 years; based on age classification by Abbott and Barnett, 2003). All animals were anesthetized with GM II and received, after loss of consciousness, an intraperitoneal injection of ketamine (400 mg/kg body weight). Bodies were transcardially perfused with cold (4°C) saline (0.9% NaCl) for 5 min. Subsequently, for fixation of the brains, cold (4°C) 4% paraformaldehyde (PFA) in 0.1 M phosphate buffer, pH 7.2, was infused for 15 min. The brains were removed and postfixed in fresh 4% PFA at 4°C. All brains had been stored in 4% PFA for variable lengths of time. Upon receipt in the laboratory, tissue was washed with 0.1 M phosphate buffered saline (PBS: 0.14 M NaCl, 2.95 mM KCl, 8.09 mM Na2HPO4, 1.47 mM KH2PO4; pH 7.4) thoroughly. Four days before sectioning, tissue was immersed in 30% sucrose in PBS and kept at 4°C.

Coronal sections (40 μm) were obtained from the medial temporal area – temporal, parietal and entorhinal cortex, and hippocampal formation (Bregma 8.00–0.80 mm according to Paxinos et al. (2012) by use of sliding microtome (Leica RM2235). All sections were immediately immersed in cryoprotectant solutions, one for light microscopy [300 g sucrose (J.T. Baker); 400 mL of 0.1M PB and 300 mL ethylene glycol (Sigma), for 1 L] and other for immunofluorescence [300 g sucrose; 10 g polyvinyl-pyrrolidone (PVP-40, Sigma); 500 mL of 0.1M PB and 300 mL ethylene glycol, for 1 L] and stored at -20°C until use.

### Immunohistochemistry

All sections were pretreated with formic acid (J.T. Baker) during 15 min and with citrate buffer 20X (Sigma) at 94°C for 10 min, except for sections incubated with anti Iba-1and AT-100. Thereafter, slides were permeabilized with 0.2% Triton X100 in PBS (0.2% PBS-triton) during 20 min. Sections were washed in PBS and incubated in 0.3% H_2_O_2_ (in PBS) for 10 min to inactivate endogenous peroxidase activity. The following washing steps were performed three times, 10 min each, in 0.2% PBS-triton. To block potential non-specific antibody binding, all sections were incubated in 5% BSA (bovine serum albumin; Sigma) in PBS for 5 min. Subsequently, sections were incubated with the following antibodies: pTau (Thr231; IgG, BioScience, Cat. No. MBS857154, 1:500), AT-100 (IgG, Jackson Immuno Research, Cat. No. 111-175-166), Aβ_1-40_ (IgG, Invitrogen, Cat. No. 44136, 1:200), Aβ_1-42_ (IgG, Invitrogen, Cat. No. 44344, 1:200), Iba-1 (IgG, Wako Chemicals, Cat. No. 019-19741, 1:300), and Alz-50 (IgM, kindly donated by Dr. Francisco García-Sierra, 1:5000) diluted in 0.2% PBS-triton.

For immunohistochemistry against tau (Phospho Thr231), Aβ_1-40_ and Aβ_1-42_, after washing, sections were incubated with biotinylated secondary antibody (IgG, Vector Laboratories, Cat. No. BA-1100, 1:500) diluted in 0.2% PBS-triton during 2 h at room temperature. Subsequently, the sections were washed and incubated with the avidin-biotin complex (ABC Kit; Vector Laboratories) in 0.2% PBS-triton for 2 h, according to the producer’s instructions. Finally, antibody binding was visualized with the chromogen 3,3′-diaminobenzidine (DAB Peroxidase Substrate Kit; Vector Laboratories) 0.025%, with 0.01% H_2_O_2_ as a catalytic agent. Control sections were processed without the primary antibody. The sections were then washed, mounted on glass slides and left to dry overnight. Dry sections were cover slipped with mounting medium Entellan (Merck).

For Alz-50 and anti-Iba-1 antibodies, sections were incubated with secondary horseradish peroxidase-conjugated antibodies (for Alz-50, anti-mouse IgM, 1:500, kindly donated by Dr. Francisco García Sierra); for anti-Iba-1 (anti-rabbit IgG, Thermo Scientific, Cat. No. 65-6120, 1:500) in 0.2% PBS-triton. Hydrogen peroxide (0.01%) and DAB (0.06%) in 0.2% PBS-triton was used to develop the horseradish peroxidase enzymatic reaction. The enzymatic reaction was stopped with 0.2% PBS-triton and then sections were mounted with Entellan (Merck) as described above.

### Immunofluorescence

Sections were pretreated with formic acid for 15 min, followed by incubation in citrate buffer 20X at 94°C for 10 min. Sections were permeabilized with 0.2% PBS-triton during 20 min. Thereafter, sections were treated with 5% BSA for 5 min, and incubated with the primary antibodies: AT-100 (IgG, Jackson Immuno Research, Cat. No. 111-175-166, 1:500), anti-Iba-1 (IgG, Wako Chemicals, Cat. No. 019-19741, 1:300), and Alz-50 (IgM, kindly donated by Dr. Francisco García-Sierra, 1:5000) in the presence of 5% horse serum (Vector Laboratories, S-2000) during 48 h (4°C). One IgG and one IgM primary antibody was used for double labeling. The sections were then washed with 0.2% PBS-triton, and incubated with secondary antibodies: FITC (anti-mouse IgM, kindly donated by Dr. Francisco García Sierra); Cy5 (anti-rabbit IgG, Jackson Immuno Research, Cat. No. 111-175-144); Cy5 (anti-mouse IgG, Jackson Immuno Research, Cat. No. 111-175-166); Alexa488 (anti-mouse IgG, Jackson Immuno Research, Cat. No. 115-545-166), with working dilution 1:500 in all cases, diluted in 0.2% PBS-triton. Control sections were processed without the primary antibody. All sections were co-incubated with DAPI (Invitrogen, 1:1000) in 0.2% PBS-triton during 30 min. The sections were then washed, mounted on glass slides. Dry sections were cover slipped with mounting medium VectaShield (Vector Laboratories).

### Image Acquisition

Nikon Eclipse 80i light microscope equipped with a Nikon DS-Ri1 camera was used to acquire bright-field images under 20× and 40× objectives, whereas for fluorescent labeling a laser scanning microscopy (Leica TCS-SP8) with argon (488 nm), and helium/neon (543 nm) lasers was used. Both lasers were always used with optimized pinhole diameter. Confocal images were obtained as z-stacks of single optical sections. Stacks of optical sections were superimposed as a single image by using the Leica LAS AF 2.6.0 build 7268 software.

### Morphometry

The immunoreactivity of phospho Thr231 (pTau231), AT-100 and Alz-50 was quantified as follows: For each subject, two brain slices (at least 800 μm apart) were imaged. Thereafter, from each slice we obtained the following images: 14 images from dorsal hippocampus (four from each CA1, CA2, and CA3, and four from dentate gyrus), eight images from entorhinal cortex (layers VI-III), 14 images from parietal (layers VI-III), and 14 images from temporal cortex (layers VI-III). The total area covered from each region was calculated as the total number of images multiplied by 276360 μm^2^ (area of a single image). We used ImageJ software (NIH, Bethesda, MD, USA) to determine the area occupied by tau aggregates. To determine the percentage of area in a determined region, the sum of the areas covered by tau aggregates was divided by the total area, and then, multiplied by 100.

For microglia quantification, iba-1 positive cells located in the dentate gyrus were analyzed. At least three slides from each age group (800 μm apart from each other) were imaged. The number of cells with different morphological state per unit area (number of cells/number of images × single image area 0.069 mm^2^) was scored in 100 ± 10 images. Based on their morphological characteristics they were classified as: resting (displaying a slight ramified morphology and small rounded soma), active (hypertrophic soma and ramified cells with extensively thick and branched processes), and dystrophic (loss of fine branches, presence of shortened tortuous processes and/or cytoplasmic fragmentation; Streit et al., 2004, 2009). The presence of cytoplasmic spheroids alone was not considered a criterion sufficient for scoring as dystrophic cells.

### Statistical Analysis

For analysis of single immunohistological markers we used one-way ANOVA, followed by a Tukey’s *post hoc* test. For microglia phenotype across ages, we used a two-way ANOVA followed by a Bonferroni’s *post hoc* test. Pearson’s correlation coefficient and probability *p*-value, which describe the significance of the correlation, were used to assess the relationships between tau hyperphosphorylation (pTau231 and AT100) and tau conformational changes (Alz50). Differences were considered statistically significant when *p* ≤ 0.05. Data are presented as means ± SEM.

## Results

### Amyloid Diffuse Plaques Were Present in Cortex of Aged Marmosets

**FIGURE 1:**
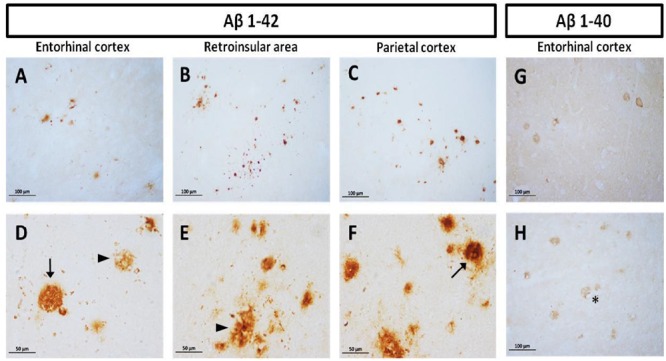
**Compact and diffuse Aβ plaques in the cortex of aged marmoset.** Aβ_1-42_ plaques in entorhinal cortex **(A,D)**, retroinsular area **(B,E)**, and parietal cortex **(C,F)**: compact (arrow in **D,F**) and diffuse plaques (arrowhead in **D,E**) are observed in cortex. Aβ_1-40_ diffuse plaques in the entorhinal **(G)** and temporal cortex **(H)**: notice the presence of Aβ_1-40_ aggregates surrounding vascular vessels (asterisk). **(A–C)** captured at 20X and **(D–F)** captured at 40X.

Diffuse plaques (without visible fibrillar Aβ or dystrophic neurites) and compact plaques (mature plaques intensely labeled with Aβ) were identified in marmoset brains, according to Dickson and Vickers (2001). We observed accumulation of Aβ_1-40_ and Aβ_1-42_ in the cortex of aged animals (entorhinal, retroinsular, and parietal cortices). A large accumulation of Aβ_1-42_ in the form of diffuse and compact plaques was found in parenchyma. No Aβ_1-42_ positive staining was observed in the hippocampal formation. Aβ_1-40_ was present only in the cortex, mainly around blood vessels as diffuse aggregates; however, it was less abundant than Aβ_1-42_ (**Figure 1**). We did not detect Aβ_1-40/1-42_ positive staining in other ages (i.e., adolescent, adult, old).

### Presence of Abnormal Hyperphosphorylation of Tau across Ontogeny of Marmoset

Using antibodies to detect phosphorylation in relevant residues of the tau aggregation process (PhosphoTau-Thr231 and AT100; Jicha et al., 1997b; Zheng-Fischhöfer et al., 1998) and conformational changes in the protein (Alz50), we detected abnormally hyperphosphorylated tau in the medial temporal area and parietal cortex of the marmosets.

#### Presence of pTau231 Hyperphosphorylated Site (Thr231)

**FIGURE 2:**
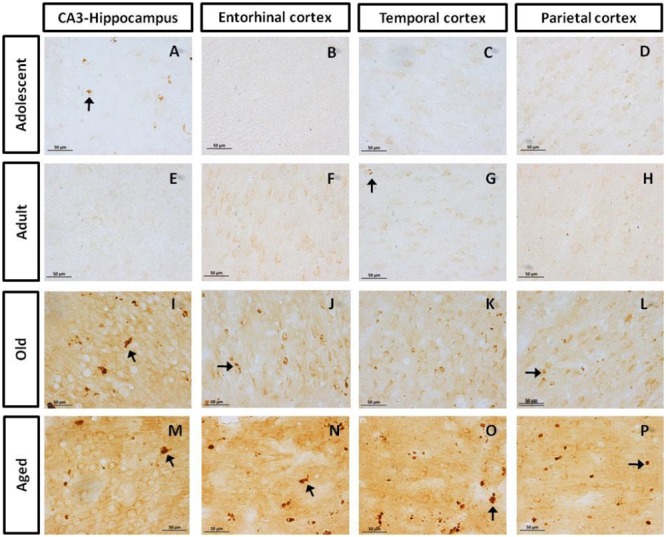
**Immunohistochemistry for pTau231 in different brain regions of the marmoset.** Adolescent subjects **(A–D)** present few pTau231 aggregates in CA3 region of the hippocampus **(A)**, but there was a lack of staining in the rest of the brain areas analyzed. In adult individuals **(E–H)** a similar labeling was observed, where few pTau231 aggregates were present in entorhinal cortex **(G)**. The old group **(I–L)** presented abundant pTau231 aggregates in all regions analyzed, and labeling was stronger in the cytoplasmic area. In aged animals **(M–P)**, pTau231 accumulates in larger and more abundant aggregates in soma and dendrites of neurons in all brain areas analyzed. Inclusions are indicated by arrows. Scale bar 50 μm.

**FIGURE 3:**
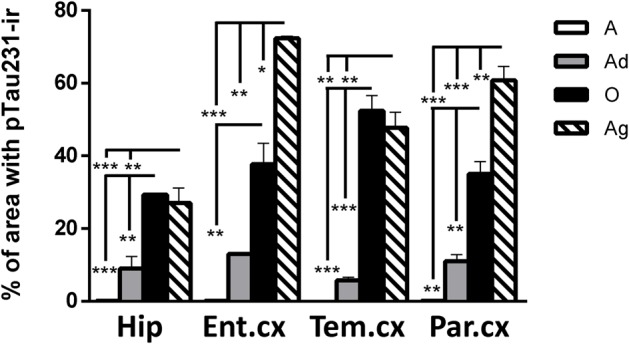
**Percentage of area occupied by pTau231-ir in hippocampus and cortices (entorhinal, temporal, and parietal) of marmoset at different ages (A, adolescents; Ad, adults; O, old; and Ag, aged).** One-way ANOVA followed by Tukey *post hoc* test. (^∗^*p* < 0.05; ^∗∗^*p* < 0.01; ^∗∗∗^*p* < 0.001).

First, we aimed to determine whether marmosets present hyperphosphorylated tau protein immunoreactive (ir) labeling in the brain (Jicha et al., 1997b). Adolescent subjects showed a light and spread pTau231-ir distribution in several regions of the medial temporal area (hippocampus, entorhinal, and temporal cortex), while adults and old individuals had a more abundant and intense labeling. In the aged subjects, pTau231-ir was heavily present in all the regions analyzed (hippocampus, entorhinal, temporal and parietal cortices; **Figure 2**). Quantification of pTau231-ir in these areas showed significant differences between old and aged subjects versus adult and adolescent groups (**Figure 3**).

#### AT100 Was Lightly Present in Adolescent and Adult Individuals, but It Increases with Aging

**FIGURE 4:**
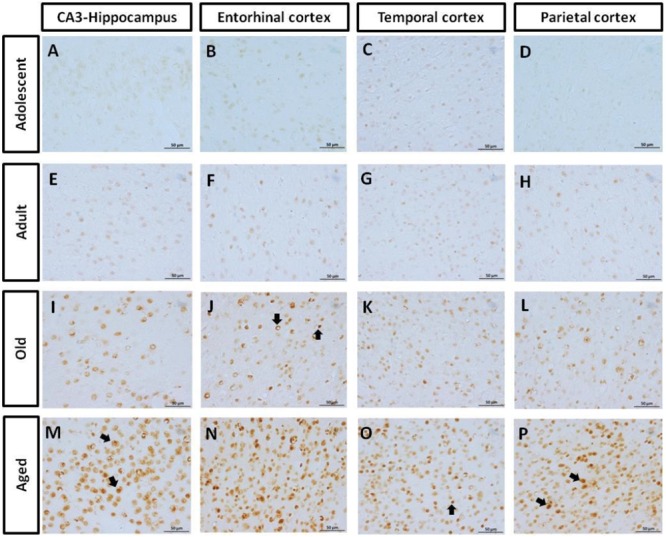
**Immunohistochemistry for AT-100 in different brain regions of marmoset.** Adolescent animals **(A–D)** presented a light phosphorylation of tau residues Thr212 and Ser214 in all regions analyzed. Adult subjects **(E–H)** showed same pattern of immunoreactivity. Old individuals **(I–L)** had a stronger labeling as AT100-ir appears in cytoplasm, but also as inclusions in the nucleus of the cells. In the aged individuals **(M–P)** AT100-ir increased in all brain regions of the brain analyzed (cytoplasmic and nuclear inclusions). Inclusions indicated by arrows. Scale bar 50 μm.

**FIGURE 5:**
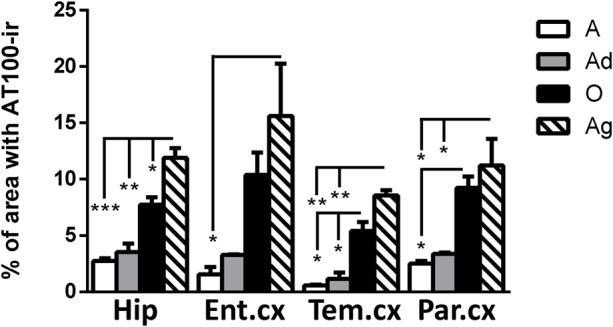
**Percentage of area occupied by AT100-ir in hippocampus and cortex (entorhinal, temporal, and parietal) of marmoset at different ages (A: adolescents, Ad: adults, O: old and Ag: aged).** One way ANOVA, *post hoc* analysis Tukey. (^∗^*p* < 0.05; ^∗∗^*p* < 0.01; ^∗∗∗^*p* < 0.001).

AT100 labels a phosphorylation site at residues Thr212 and Ser214 (Zheng-Fischhöfer et al., 1998). In the brain of marmosets, AT100-ir was detected as aggregates in the cytoplasm in all regions at all ages. In contrast, larger AT100-ir inclusions were observed in old and aged marmosets (**Figure 4**). Further quantification analysis indicated a significant increase of AT100-ir in old and aged animals, versus those adolescent and adult (**Figure 5**).

#### Conformational Changes of Tau Protein Were Detected in All Subjects but Increase with Aging

Alz50 binds to two discontinuous sequences of tau, one consisting of residues 5–15 at N-terminus and another in the residues 312–322 of the third microtubule-binding repeat domain (Carmel et al., 1996; Jicha et al., 1997a). Alz50-ir depends on the self-folding of N-terminus onto the third microtubule-binding repeat domain, thus allowing the recognition of early aggregation of tau protein (Weaver et al., 2000).

**FIGURE 6:**
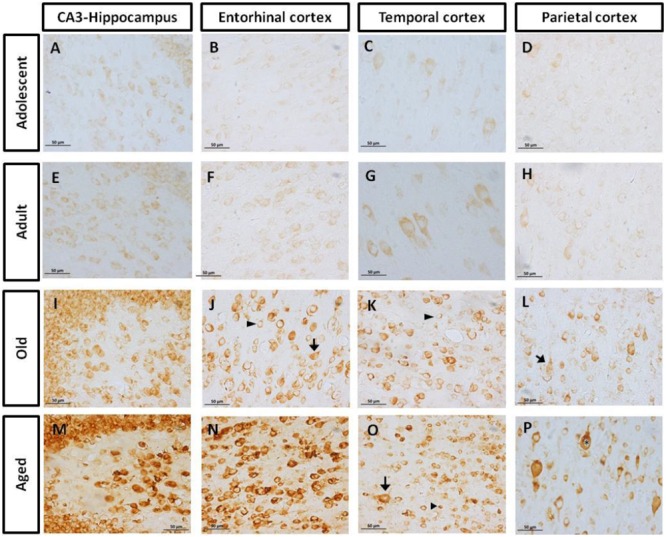
**Immunohistochemistry for Alz50 in different brain regions of marmoset.** Adolescent subjects **(A–D)** present a faint Alz50 positive labeling. In adults **(E–H)** a similar pattern was observed, however, with stronger immunoreactivity. Old subjects **(I–L)** presented an intense and abundant Alz50-ir in neurons (arrow) and glia cells (arrowhead). In aged subjects **(M–P)**, Alz50-ir was strongly present in all brain regions, where also dendrites were dense labeled. Fibrillary inclusions are observed in neurons (arrow) and glia cells (arrowhead). Fibrillary inclusions are observed in principal like-cells (^∗^). Scale bar 50 μm.

**FIGURE 7:**
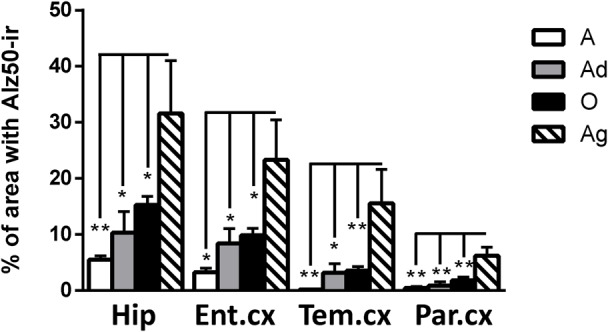
**Percentage of immunoreactivity for Alz50 in hippocampus and cortex (entorhinal, temporal, and parietal) of marmoset at different ages (A: adolescents, Ad: adults, O: old, and Ag: aged).** One way ANOVA, *post hoc* analysis Tukey. (^∗^*p* < 0.05; ^∗∗^*p* < 0.01).

Our results show Alz50-ir in adolescent and adult subjects as a faint staining mainly in cytoplasmic compartments (**Figures 6A–H**). In old subjects, a stronger Alz50-ir was located in cytoplasm mainly in a fibrillary form. Moreover, Alz50-ir was present in neurons with a principal cell-like-morphology, but also in structures that resemble glial cells (**Figures 6I–L**). Aged individuals had stronger Alz50-ir with cytoplasmic fibrillary inclusions and labeling of dendrites of principal neurons, and a dense Alz50-ir in glia like-cells (**Figures 6M–P**). Statistical analysis showed a significant increase of Alz50-ir in aged animals compared to younger ones (300% increase compared to old, and over 800% increase compared to adolescent subjects; **Figure 7**).

#### Correlation of Tau Hyperphosphorylation versus Conformational Change in Marmoset Brain

**FIGURE 8:**
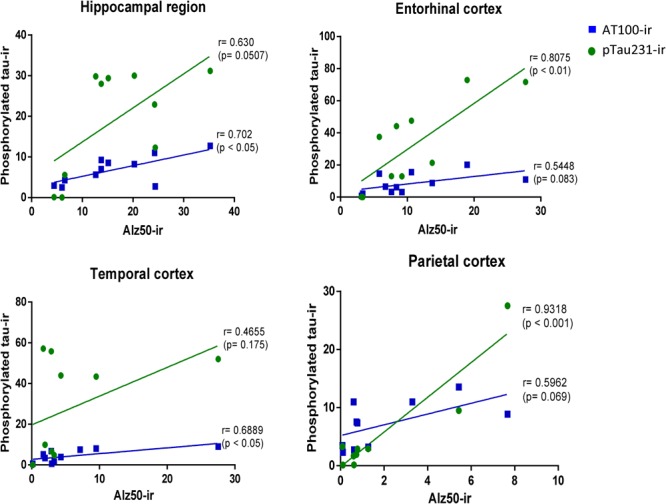
**Correlation analysis of phosphorylated tau markers versus Alz-50-ir in the hippocampal area and entorhinal, temporal, and parietal cortices.** Pearson correlation (r) was used for statistical analysis; *p* indicates significant differences.

In order to determine the strength of the association of markers of hyperphosphorylation with those related to conformational changes of tau protein, Pearson correlation was used. In all regions analyzed, there was a positive correlation between pTau231/AT100 with Alz-50-ir: pTau231-ir was significantly correlated to Alz50-ir in entorhinal (*r* = 0.807, *p* < 0.01) and parietal (*r* = 0.931, *p* < 0.001) cortices whereas AT100-ir and Alz50-ir were significantly correlated in hippocampus (*r* = 0.702, *p* < 0.05) and temporal cortex (*r* = 0.688, *p* < 0.05). The slope of the linear regression was in all cases higher for pTau231-ir than for AT100 (**Figure 8**). Moreover, when tau markers where plotted against age, a similar pattern was observed. PTau231 presented the highest correlation in entorhinal (*r* = 0.934, *p* > 0.0001) and parietal (*r* = 0.936, *p* > 0.0001) cortices compared to AT100-ir and Alz50-ir. However, in hippocampus and temporal cortex, although pTau231-ir was higher than AT100-ir, correlation analysis indicated a stronger association of AT100-ir (*r* = 0.834, *p* > 0.01 and *r* = 0.873, *p* > 0.001, respectively) with age (**Supplementary Figure S1**).

### Morphological Alterations of Microglia Cells in Marmosets

#### Changes in Microglia Phenotype with Aging

**FIGURE 9:**
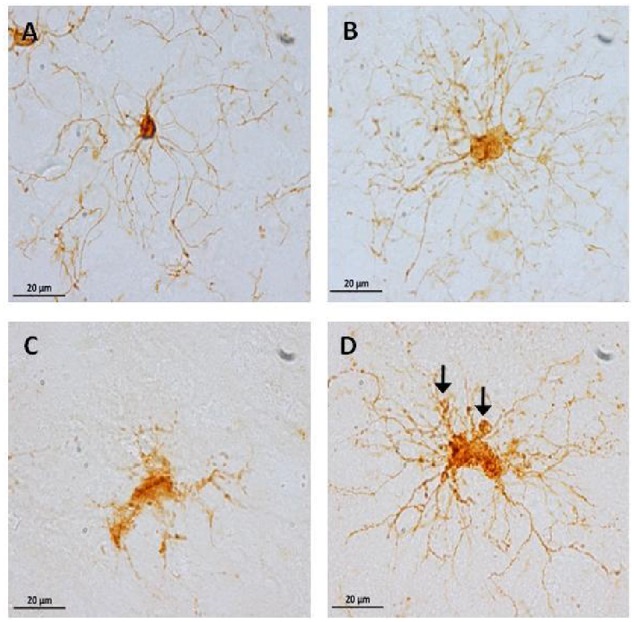
**Representative photomicrographs of microglia phenotypes: inactive cells** (**A**, from an adolescent subject) with a small soma size, and long and thin processes. Active cells (**B**, from an old subject) with hypertrophy and abundant processes. Dystrophic cells (**C,D**, from an aged subject) with clear fragmentation of the cytoplasm and de-ramification **(C)**, diverse spheroids and shortened tortuous process (**D**, arrows).

**FIGURE 10:**
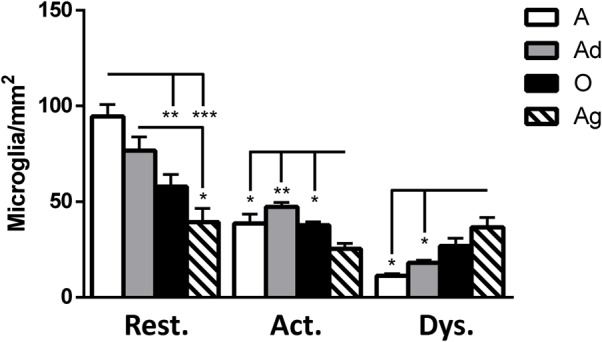
**Quantification of resting, active and dystrophic microglia per area (mm^2^) in dorsal hippocampus of marmosets at different ages (A: adolescents, Ad: adults, O: old, and Ag: aged).** One way ANOVA, *post hoc* analysis Tukey (^∗^*p* < 0.05; ^∗∗^*p* < 0.01; ^∗∗∗^*p* < 0.001).

It has been described that microglia are subject to different morphological alterations related to their active state during aging and neurodegeneration (von Eitzen et al., 1998; Streit et al., 2004, 2009; Simmons et al., 2007; Xue and Streit, 2011). Microglial activation can lead to uncontrolled or prolonged neuroinflammation, a potentially harmful event that can result in cellular damage. Prolonged microglia activation also leads to the loss of function and a dystrophic phenotype (Streit et al., 2004). We focuses our study on the classification microglia as resting, active, or dystrophic state, based on previous studies (Streit et al., 2004, 2009). The visualization of microglia with anti-iba1 clearly shows these cells under resting state (characterized by round soma and long extended processes), active (characterized by hypertrophy of the soma and processes), and dystrophic state (characterized by abnormal cytoplasm, fragmented and helicoidally process; **Figure 9**). We then sought to determine the microglia’s phenotype in the dorsal hippocampal region of adolescent, adult, old, and aged individuals. The total number of microglia was similar among all ages (range: 101–156 cells; **Figure 10**). However, the morphological classification indicates an age-dependent phenotype. Resting microglia was the most prominent phenotype across ages, being higher in adolescent animals compared to old and aged subjects (**Figure 10**). Active microglia presented a more homogeneous presence in all ages, but it was significantly decreased in the aged subjects compared to adolescent, adult, and old animals (**Figure 10**). Dystrophic microglia revealed the lowest frequency across the ontogeny; however, it was higher in the aged animals compared to adolescent and adult animals (**Figure 10**).

#### Dystrophic Microglia Presented Hyperphosphorylation and Conformational Changes in Tau

**FIGURE 11:**
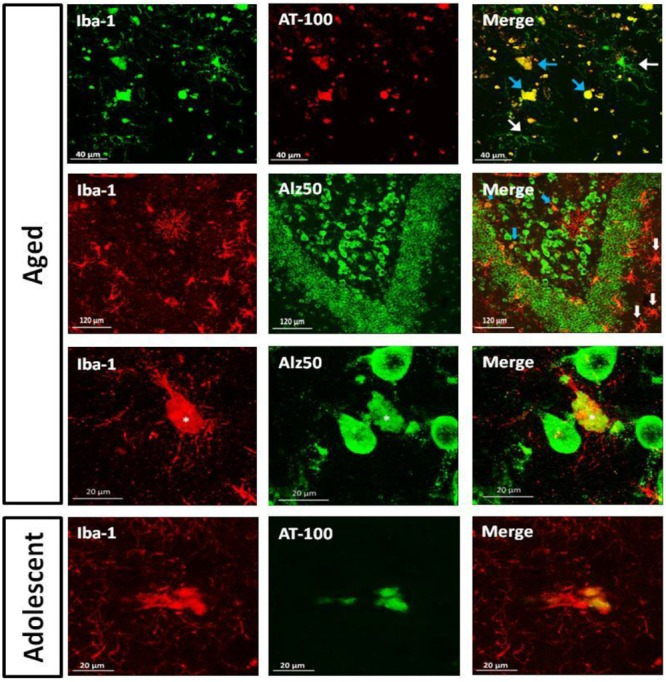
**Double labeling of microglia (iba-1) and tau hyperphosphorylation (AT-100) or conformational change (Alz50) in dentate gyrus.** Aged marmoset: Dystrophic microglia presented compact AT100-ir aggregates (blue arrows), but active microglia did not present any AT100-ir (white arrows). In the polymorphic layer dystrophic microglia show Alz50-ir as large cytoplasmic inclusions (blue arrows), whereas in the molecular layer, active microglia (white arrows) do not present any Alz50-ir. A magnification of the polymorphic layer showed dystrophic microglia with fragmented processes and Alz50-ir (^∗^), while neighboring cells were not positive for iba-1. Adolescent marmoset: Dystrophic microglia present AT100-ir as diffuse inclusions.

As we detected the presence abnormal tau hyperphosphorylation in structures that resembled glia cells (**Figures 4** and **6**), we aimed to segregate the presence of Alz50-ir and AT100-ir regarding the microglia’s phenotype. We focused on the dentate gyrus as this area presented a large amount of dystrophic microglia. In the aged animals, AT100 was present in every dystrophic cell as dense inclusions, but active microglia did not show AT100-ir (**Figure 11**). In addition, Alz50-ir was highly present in dystrophic microglia, while active microglia did not present any labeling (**Figure 11**).

As we observed few dystrophic microglia cells in adolescent and adult marmosets (**Figure 10**), we sought to determine whether dystrophic microglia in this young animals also contain abnormal tau or whether it was exclusive of aged individuals. In adolescents there were very few dystrophic cells, but those were also positive for AT100; although AT100-ir was more diffuse compared to aged animals. Active and resting microglia did not show AT100-ir (**Figure 11**).

## Discussion

### Amyloid Plaques Are Present in Aged Marmoset, but Abnormal Tau Can Be Detected from Adolescence

Aβ is a small peptide involved in the pathogenesis of AD in humans (Zhang et al., 2012), either in the form of compact or diffuse amyloid plaques (Blennow et al., 2006). Compact plaques present an amyloid core while diffuse plaques lack a morphologically identifiable structure and are characterized by deposits in the parenchyma and around wall vessels (Dickson and Vickers, 2001). Monkeys have identical Aβ amino acid sequence to humans (Selkoe et al., 1987), which may result in similar mechanisms for Aβ production and accumulation. It has widely been reported that aged non-human primates present amyloid accumulation in the form of diffuse and compact plaques, mainly in the cortex (Cork et al., 1990; Gearing et al., 1996, 1997; Giannakopoulos et al., 1997; Kimura et al., 2003; Bons et al., 2006; Lemere et al., 2008; Oikawa et al., 2010; Perez et al., 2013; Sridharan et al., 2013; Darusman et al., 2014). Amyloid plaques in the cortex of old marmoset have been previously described as well (Maclean et al., 2000; Geula et al., 2002). In the present study we observed Aβ_1-40_ and Aβ_1-42_ accumulation in the medial temporal cortex and adjacent cortical areas of aged marmosets. We observed Aβ_1-42_ plaques, mainly in the form of diffuse plaques, but some large compact plaques were also detected in the parenchyma. Aβ_1-40_ diffuse plaques were present mainly around blood vessels as already described for cerebral amyloid angiopathy (CAA) in humans (Boulouis et al., 2016); however, Aβ_1-40_ compact plaques were not observed. No Aβ staining was observed in younger animals.

Diffuse plaques can be detected in cognitively intact elderly people (Mackenzie, 1994; Hof et al., 1996; Mufson et al., 1999; Rodrigue et al., 2009), and it has been described that they do not trigger any changes in the neuropil, such as; increase in neurite curvature, dystrophic neurites, and recruitment of astrocytes (Masliah et al., 1990; Lombardo et al., 2003). Furthermore, extracellular diffuse deposits can be detected in the postmortem brain of Down’s syndrome patients already at 12 years of age whereas compact plaques are only seen after the third decade, along with cognitive deficits in Down syndrome subjects (Lemere et al., 1996). Thus, it has been postulated that diffuse plaques represent an early stage in AD plaque development whereas compact plaques are associated with late AD stages and cognitive decline (Dickson and Vickers, 2001). In our present study, Aβ_1-42_ was detected as diffuse and compact plaques in the aged marmoset. We also observed tau hyperphosphorylation in aged marmoset. Thus, aged marmoset presents two main hallmarks of neurodegenerative diseases as AD: compact amyloid plaques and abnormal tau phosphorylation.

Alzheimer’s disease is considered a multifactorial disease that progresses over time (Iqbal and Grundke-Iqbal, 2010) where brain alterations can be detected decades before clinical symptoms of the disease. Accordingly, it has been postulated that one pathogenic trigger of neuronal dysfunction comprises soluble Aβ oligomers, long before formation of amyloid plaques (Lacor et al., 2004). Soluble Aβ_1-42_ is considered to be highly neurotoxic as it can induce mitochondria dysfunction (Cha et al., 2012), spine loss (Yu and Lu, 2012), membrane fluidity (Sasahara et al., 2013), and over-excitation of the post-synaptic neurons (Palop et al., 2007), leading to impaired memory and cognition (Sengupta et al., 2016). In this study we could not measure the amount of soluble Aβ as no fresh tissue was available. Therefore, further studies are needed to determine the presence of soluble Aβ species in brain and plasma samples, rather than the fibrillary forms already reported in literature, to better outline the age of appearance of this peptide in marmosets.

Abnormally phosphorylated tau protein is a hallmark of several human neurodegenerative disorders (García-Sierra et al., 2003; Binder et al., 2005; Ferrer et al., 2014). However, phosphorylation of tau may also occur under physiological processes. Physiological based phosphorylation of tau allows microtubules to disassemble (Lindwall and Cole, 1984), a phenomenon observed during hibernation (Arendt et al., 2003) or cellular division (Delobel et al., 2002). Notwithstanding, in aging (Hof et al., 1996) and neurodegenerative diseases (i.e., AD, Down syndrome, and tauopathies) an excessive phosphorylation of tau causes its self-aggregation (Alonso et al., 2001) in straight and paired-helical filaments which subsequently form the so called neurofibrillary tangles (NFT). NFTs cause neuronal dysfunction and eventually lead to death (Avila et al., 2006; Stokin and Goldstein, 2006). In several aged non-human primates, hyperphosphorylated tau filaments have been observed in hippocampus and cortex of old subjects (Härtig et al., 2000; Schultz et al., 2000a,b; Oikawa et al., 2010; Perez et al., 2013; Darusman et al., 2014). To our knowledge, there are no previous reports of hyperphosphorylated tau in marmosets. In this study, adolescent animals (1.6–5 years of age) already showed hyperphosphorylated tau (AT-100 and pTau231), while old subjects (>8 years-old) had a dramatically increased immunolabeling in parietal, temporal, and entorhinal cortices, and in the hippocampus. Also, conformational changes in tau (Alz50-ir) were detected from adolescence, and increased with age. Our results coincide with the study of Perez et al. (2013) in *Gorilla gorilla*, where Alz50-ir fibers were observed at each age analyzed (13–55 years of age). In *Papio anubis* (Schultz et al., 2000b), *Papio hamadryas* (Schultz et al., 2000a), *Macaca fascicularis* (Oikawa et al., 2010; Darusman et al., 2014), and *Macaca mulatta* (Härtig et al., 2000) hyperphosphorylated tau appears until the second decade of life. This could suggest that the beginning of tau hyperphosphorylation process varies between different species of non-human primates, the marmosets being heavily prone to be affected.

In order to better understand the association between the hyperphosphorylation of tau and its conformational changes, we performed a correlation analysis between pTau231/AT100 and Alz-50 for each marmoset (all ages). We observed that both markers increase proportionally to each other in most of the brain regions; however, pTau231 showed a better correlation index in the entorhinal and parietal cortices (**Figure 8**). In AD, the spread of tau pathology from entorhinal cortex to the limbic region (hippocampus) takes several years to progress (Braak and Braak, 1991). Therefore, in agreement with human studies, abnormal tau phosphorylation was higher in entorhinal cortex than in other brain areas, and progresses over the years to hippocampal region in the marmoset brain (**Figure 3**).

Conformational changes of tau were detected by Alz50 antibody. When, tau markers were correlated with age, Alz50 presented the lowest correlation index as it increased only in the aged marmosets whereas pTau231 and AT100 increased constantly since adolescence (**Supplementary Figure S1**). A recent analysis of 2,332 brains of 1- to 100-year-old individuals, clearly showed that, in humans, hyperphosphorylation of tau commences before puberty or in early adulthood and accumulates along aging until NFTs can be observed (Braak et al., 2011). Moreover, previous studies have described that hyperphosphorylation of tau must precede the appearance of tau aggregates and/or its conformational changes (as detected by Alz-50; Alonso et al., 2001; García-Sierra et al., 2003). This previous reports are in accordance to our present observation where tau hyperphosphorylation was more abundant than tau conformational changes in the marmoset. Interestingly, the distribution patterns of amyloid plaques were different from the one of tau pathology (amyloid plaques were found only in cortex, while abnormal tau was present in cortex and hippocampus), suggesting that these lesions develop independently from each other. Then, in the marmoset, Aβ deposition is not a necessary precondition for hyperphosphorylation of tau, as already suggested by other authors (Iqbal and Grundke-Iqbal, 2010; Iqbal et al., 2014).

### Dystrophic Microglia in Marmoset

In murine models of AD, amyloid plaques formation is followed by the appearance of activated microglia. Streit et al. (2004) argued that in aged humans, microglia normally senesce and undergo microglial dystrophy that, in some cases, involves a process of cytorrhexis. While this latter process can be observed in normal aging brains, it is more frequently observed in neurodegenerative diseases, such as AD (Streit et al., 2004, 2009). In this study, we classified the morphology of microglia as inactive, active, and dystrophic; and quantified them according to the age. There was a decreased number of resting microglia along aging. Active cells showed a more homogeneous presence in all ages, while dystrophic microglia increased in aged animals compared to adolescent and adults. These results coincide with data from patients suffering neurodegenerative diseases where there is a pronounced increase of dystrophic microglia (AD, Huntington’s Disease, Creutzfeldt-Jakob disease; von Eitzen et al., 1998; Wierzba-Bobrowicz et al., 2004; Simmons et al., 2007; Streit et al., 2009; Xue and Streit, 2011), highlighting the role of microglia in neurodegenerative diseases. It is important to mention that, in contrast to AD where active microglia proliferate around amyloid plaques, dystrophic microglia in the marmoset was found in brain of adolescent and adult marmoset without apparent amyloid deposition.

### Abnormal Tau Is Present Only in the Dystrophic Microglia

Notably, in the present study dystrophic microglia was Alz50-ir (widely spread in cytoplasm), and AT100-ir positive (in the form of inclusions). This pattern of immunoreactivity was observed in both the few dystrophic cells found in adolescent individuals and the large amount of dystrophic cells found in the aged animals. In contrast, active microglia were always negative for Alz50 and AT100 in all ages tested.

The mechanisms leading to elevated tau hyperphosphorylation and its aggregation in neurodegenerative diseases still remain unclear. On the one side, neuroinflammation has been implicated in driving hyperphosphorylation and aggregation of tau and neurodegeneration in humans (Gebicke-Haerter, 2001; Ishizawa and Dickson, 2001; Bellucci et al., 2011) and various models of tauopathies (Bellucci et al., 2004; Zilka et al., 2009). In the 3xTg mouse model of AD, induction of systemic inflammation with lipopolysaccharide resulted in enhanced microglial activation and tau pathology (Kitazawa, 2005). The microglial fractalkine receptor (CX3CR1) knockout mice (hTauCx3cr1-/-) showed enhanced microglia-specific neuroinflammation, accompanied by an accelerated onset and progression of tau pathology, cognitive dysfunction and neurodegeneration (Maphis et al., 2015). Furthermore, microglial activation preceded tau pathology and synaptic loss in the P301S mouse model of tauopathy, while administration of FK506, an anti-inflammatory compound, reduced tau pathology and prolonged the lifespan of these mice (Yoshiyama et al., 2007).

On the other hand, more recent research proposes a neuroprotective role of microglia. Increased intracellular levels of phosphorylated tau could be detrimental to neurons. Thus, tau secretion might be a mechanism by which excess of neuronal tau is removed to prevent toxicity. The spread of tau from cell to cell has been proposed to be one of the mechanisms underlying the progression of tau pathologies (Holmes et al., 2013, 2014). Recent studies demonstrate that microglia are involved in the uptake of tau protein. In primary cultures of microglia (Luo et al., 2015; Bolós et al., 2016) and P301S mice (Luo et al., 2015), microglia internalize extracellular soluble and insoluble tau *in vitro* and *in vivo*, respectively. Thus, microglia seem to play a protective mechanism in the clearance of tau. Furthermore, a deficiency in the capacity of microglia to internalize and degrade extracellular toxic proteins might be related to a higher incidence of neurodegenerative disease. Among the newly identified AD-risk genes, many are important regulators of innate immunity, including the antigen-presenting and phagocytic and degradative functions of circulating macrophages and microglia (Griciuc et al., 2013; Guerreiro et al., 2013; Borroni et al., 2014; Kleinberger et al., 2014). An arginine-to-histidine substitution at amino acid 47 (R47H) in the triggering receptor expressed on myeloid cells 2 (TREM2) gene, which regulates microglial function in the CNS, increased the risk of developing late-onset AD (Guerreiro et al., 2013).

Promoting the uptake of tau aggregates into microglia could be of great importance in the development of more effective therapies against AD and other tauopathies. In mouse models of tau-dependent neurodegeneration, passive immunization with anti-tau monoclonal antibodies has been shown to reduce age-dependent tau pathology, including NFTs, neurodegeneration and behavioral impairment (Asuni et al., 2007; Bi et al., 2011; Boutajangout et al., 2011; Chai et al., 2011; Yanamandra et al., 2013). Furthermore, two different antibodies (HJ8.5 and HJ9.4) were able to increase tau clearance via microglia and a parallel block of tau uptake into neurons, in a size-dependent manner (Funk et al., 2015). Still, whether microglia will undergo neurodegeneration after tau uptake or whether these cells will actively function in later stages remains unclear.

In the present study, the high amounts of hyperphosphorylated tau observed in the dystrophic microglia of aged marmoset could suggest that long termed tau-phagocytosis triggers the dystrophic process since hyperphosphorylated tau appears early in the adolescence.

In summary, marmosets seem to be a valuable prospect species for aging research as they present important hallmarks of human brain aging and neurodegeneration.

## Ethics Statement

All animal experiments were approved by the Lower Saxony Federal State Office for Consumer Protection and Food Safety, Germany

## Author Contributions

JR-C: Substantially contributed in the acquisition, analysis, and interpretation of data for the work; and contributed in the drafting the work. Finally approved the version to be published, and agree to be accountable for all aspects of the work. EF: Substantial contributed in the interpretation of data for the work; revising it critically for important intellectual content. He has given final approval of the version to be published; and agreed to be accountable for all aspects of the work. CP-C: Substantial contributed to the conception, design, and interpretation of data for the work; drafting the work and revising it critically for important intellectual content. She has finally approved the version to be published; and agreed to be accountable for all aspects of the work in ensuring that questions related to the accuracy or integrity of any part of the work are appropriately investigated and resolved.

## Conflict of Interest Statement

The authors declare that the research was conducted in the absence of any commercial or financial relationships that could be construed as a potential conflict of interest.

## Supplementary Material

FIGURE S1 **Correlation analysis of abnormal tau markers versus age in the hippocampal area, and entorhinal-, temporal-, and parietal cortices.** Pearson correlation (r) was used for statistical analysis; *p* indicates significant differences.
