# Corrigendum: Evidence of Tau Hyperphosphorylation and Dystrophic Microglia in the Common Marmoset

**DOI:** 10.3389/fnagi.2017.00032

**Published:** 2017-02-21

**Authors:** Juan D. Rodriguez-Callejas, Eberhard Fuchs, Claudia Perez-Cruz

**Affiliations:** ^1^Laboratory of Neuroplasticity and Neurodegeneration, Department of Pharmacology, Center for Research and Advanced Studies (CINVESTAV)Mexico City, Mexico; ^2^Clinical Neurobiology Laboratory, German Primate Center – Leibniz Institute for Primate ResearchGöttingen, Germany

**Keywords:** aging, neurodegeneration, animal models, non-human primate

In the original article, we neglected to thank Dr. Lester Binder for the donation of the Alz-50 antibody. The authors apologize for this oversight. This error does not change the scientific conclusions of the article in any way.

## Conflict of interest statement

The authors declare that the research was conducted in the absence of any commercial or financial relationships that could be construed as a potential conflict of interest.

